# Draft Genome Sequence of an Environmental Vibrio cholerae Strain, 2012Env-25, Obtained Using Nanopore Sequencing Technology

**DOI:** 10.1128/MRA.00625-20

**Published:** 2020-08-06

**Authors:** V Lyju Jose, Matthew T. Pileggi, Meer Taifur Alam, Afsar Ali, Adam C. N. Wong

**Affiliations:** a Entomology and Nematology Department, University of Florida, Gainesville, Florida, USA; b Genetics Institute, University of Florida, Gainesville, Florida, USA; c Emerging Pathogens Institute, University of Florida, Gainesville, Florida, USA; d Department of Environmental & Global Health, College of Public Health & Health Professions, University of Florida, Gainesville, Florida, USA; University of Maryland School of Medicine

## Abstract

Vibrio cholerae is a halophilic Gram-negative bacterial species and the etiological agent of cholera. Here, we report the draft genome sequence of an environmental V. cholerae strain, 2012Env-25, obtained using Oxford Nanopore Technologies (ONT) to provide insights into the ecology, evolution, and pathogenic potential of this bacterium.

## ANNOUNCEMENT

Toxigenic Vibrio cholerae is a Gram-negative bacterium and the causative agent of cholera, a profuse and acute watery diarrheal disease in humans (1, 2). Much of the research focus has been on the toxigenic V. cholerae strains of the O1/O139 serogroups, as they are responsible for all cholera pandemics. However, there is a diversity of non-O1/non-O139 serogroups that can cause sporadic infections in humans. The ecology and evolution of V. cholerae in the environment are also poorly understood (3). To assess the impact and trajectory of cholera in Haiti, the University of Florida Emerging Pathogens Institute (EPI) and College of Public Health & Health Professions established a fully functional field microbiology laboratory in the rural Gressier region of Haiti in November 2011. Beginning in May 2012, we established a monthly environmental survey for the isolation and identification of toxigenic V. cholerae from 17 fixed sites, including river and estuarine sites. We isolated and characterized a nontoxigenic V. cholerae O1 strain designated 2012Env-25 from a site (La Salle, Haiti) in April 2012. The water samples were streaked onto thiosulfate citrate bile salts (TCBS) sucrose agar plates and incubated overnight at 37°C. The distinct colonies were transferred to Luria agar plates for further screening and molecular characterization (4). Individual colonies were grown in LB broth at 37°C overnight for genomic DNA isolation.

The bacterial cells were pelleted at 12,000 × *g* for 5 min and homogenized in lysis buffer (20 mM Tris Cl [pH 8.0], 2 mM sodium EDTA, 1.2% Triton X-100, and 20 mg/ml lysozyme) using a bead beater (Bertin Instruments), and the genomic DNA was isolated using the salting-out protocol as described in a previous study (5). The genomic library was prepared using the ligation sequencing kit 1D (SQK-LSK108) as per the manufacturer’s instruction, and the DNA was not sheared or size selected. Whole-genome sequencing was performed on an Oxford Nanopore Technologies MinION Mk1b device using a FLO-MIN106 (R9) flow cell with MinKNOW v19.10.1 software at the Entomology and Nematology laboratory, University of Florida. The raw sequence files were base called by Guppy v3.4.1, and the low-quality reads were removed from further analysis. Adapters and chimeric sequences were removed using Porechop v0.2.4 (https://github.com/rrwick/Porechop). Trimming, correction, and assembly were carried out by Canu v1.9 (6), and the sequences were then aligned using minimap2 v2.15-r914-dirty (7). The bacterial genome assembly obtained was polished using Nanopolish v0.10.1 (8). The quality of the genome assembly was assessed using QUAST v5.0.2 (9). The final polished genome was annotated using NCBI Prokaryotic Genome Annotation Pipeline (PGAP) v4.11 (10). The software default parameters were used except where otherwise noted.

Oxford Nanopore Technologies (ONT) generated a total of 17,901 reads, accounting for 151,833,237 bases with a mean length of 8,551 bp and 38× coverage. The largest read was 86,745 bp long. After read correction and trimming, the Canu assembler v1.9 generated 2 contigs representing the two different circular chromosomes of the V. cholerae genome. The overlapping chromosome regions from the final assembly were removed manually. The first contig representing the first chromosome was larger in size (2,984,828 bp) than the second contig (1,115,776 bp) representing the other chromosome, and the total length of the assembly was 4,100,604 bp. The quality check of assembly using QUAST v5.0.2 indicated that the genome assembly had 89.97% of genome fraction. The GC content of the genome was 47.72%, with a read *N*_50_ value of 2,984 kb. The PGAP annotation identified 3,872 coding DNA sequences and 140 RNA sequences in the assembly (105 tRNAs, 31 complete rRNAs, and 4 noncoding RNAs [ncRNAs]). ONT produced the near-full-length genome of V. cholerae, and 3,188 genes with frameshifts were identified.

### Data availability.

This whole-genome shotgun project has been deposited at DDBJ/ENA/GenBank under the accession numbers CP053555 and CP053556. The raw sequencing reads have been deposited in the NCBI Sequence Read Archive (SRA) under accession number SRR11838355, and the fast5 files are available under the accession number SRP263209.
